# Content and User Engagement of Health-Related Behavior Tweets Posted by Mass Media Outlets From Spain and the United States Early in the COVID-19 Pandemic: Observational Infodemiology Study

**DOI:** 10.2196/43685

**Published:** 2023-08-22

**Authors:** Miguel Angel Alvarez-Mon, Victor Pereira-Sanchez, Elizabeth R Hooker, Facundo Sanchez, Melchor Alvarez-Mon, Alan R Teo

**Affiliations:** 1 Department of Medicine and Medical Specialties Faculty of Medicine and Health Sciences University of Alcala Alcala de Henares Spain; 2 Department of Psychiatry and Mental Health University Hospital Infanta Leonor Madrid Spain; 3 Ramón y Cajal Institute of Sanitary Research (IRYCIS) Madrid Spain; 4 Department of Child and Adolescent Psychiatry NYU Grossman School of Medicine New York, NY United States; 5 VA Portland Health Care System, Health Services Research & Development Center to Improve Veteran Involvement in Care Portland, OR United States; 6 OHSU-PSU School of Public Health Oregon Health and Science University Portland, OR United States; 7 Lincoln Medical and Mental Health Center New York, NY United States; 8 Devers Eye Institute Portland, OR United States; 9 Department of Psychiatry, Oregon Health & Science University Portland, OR United States

**Keywords:** COVID-19, health communication, social media, Twitter, health promotion, public health, mass media

## Abstract

**Background:**

During the early pandemic, there was substantial variation in public and government responses to COVID-19 in Europe and the United States. Mass media are a vital source of health information and news, frequently disseminating this information through social media, and may influence public and policy responses to the pandemic.

**Objective:**

This study aims to describe the extent to which major media outlets in the United States and Spain tweeted about health-related behaviors (HRBs) relevant to COVID-19, compare the tweeting patterns between media outlets of both countries, and determine user engagement in response to these tweets.

**Methods:**

We investigated tweets posted by 30 major media outlets (n=17, 57% from Spain and n=13, 43% from the United States) between December 1, 2019 and May 31, 2020, which included keywords related to HRBs relevant to COVID-19. We classified tweets into 6 categories: mask-wearing, physical distancing, handwashing, quarantine or confinement, disinfecting objects, or multiple HRBs (any combination of the prior HRB categories). Additionally, we assessed the likes and retweets generated by each tweet. Poisson regression analyses compared the average predicted number of likes and retweets between the different HRB categories and between countries.

**Results:**

Of 50,415 tweets initially collected, 8552 contained content associated with an HRB relevant to COVID-19. Of these, 600 were randomly chosen for training, and 2351 tweets were randomly selected for manual content analysis. Of the 2351 COVID-19–related tweets included in the content analysis, 62.91% (1479/2351) mentioned at least one HRB. The proportion of COVID-19 tweets mentioning at least one HRB differed significantly between countries (*P*=.006). Quarantine or confinement was mentioned in nearly half of all the HRB tweets in both countries. In contrast, the least frequently mentioned HRBs were disinfecting objects in Spain 6.9% (56/809) and handwashing in the United States 9.1% (61/670). For tweets from the United States mentioning at least one HRB, disinfecting objects had the highest median likes and retweets, whereas mask-wearing– and handwashing-related tweets achieved the highest median number of likes in Spain. Tweets from Spain that mentioned social distancing or disinfecting objects had a significantly lower predicted count of likes compared with tweets mentioning a different HRB (*P*=.02 and *P*=.01, respectively). Tweets from the United States that mentioned quarantine or confinement or disinfecting objects had a significantly lower predicted number of likes compared with tweets mentioning a different HRB (*P*<.001), whereas mask- and handwashing-related tweets had a significantly greater predicted number of likes (*P*=.04 and *P*=.02, respectively).

**Conclusions:**

The type of HRB content and engagement with media outlet tweets varied between Spain and the United States early in the pandemic. However, content related to quarantine or confinement and engagement with handwashing was relatively high in both countries.

## Introduction

Harnessing Twitter during the COVID-19 pandemic may have been beneficial [1,2]. In previous disasters (eg, H1N1 outbreak), Twitter was among the most used social platforms [3-5]. Mass media outlets are a key tool during disasters such as the COVID-19 pandemic because they can educate people [6]. Through Twitter alone, millions of users interact each day, and during disasters the number of interactions and tweets increases [7]. Tweets during disasters can inform the public of risk factors, where to ask for help, locations and availability of hospitals, and locations of people who need help (eg, elderly living alone) [8]. If media outlets share accurate and valuable information (eg, encourage people to stay at home) through Twitter, they may contribute to saving lives [9]. Mass media outlet Twitter accounts can become an excellent resource for the public to stay updated on health policies during the COVID-19 pandemic [10].

Some behavioral measures, such as handwashing, mask-wearing, and physical distancing, are among the most effective tools to combat the COVID-19 pandemic [11,12]. These are considered preventive health behaviors. The World Health Organization, the Centers for Disease Control and Prevention, and many other health institutions have addressed their importance in slowing the spread of the coronavirus. Furthermore, shelter in place (stay-at-home orders for the general population), quarantine (separation and restriction of movements of people who have potentially been exposed to COVID-19), and isolation (separation of people who have been diagnosed from people who are not sick) have been mandatory in many countries around the world.

Social media can be used to create indicators of the health environment that are associated with area-level mortality and health behaviors [13,14]. Local area characteristics are increasingly associated with health outcomes. Social processes affect health through the maintenance of social norms, stimulation of new interests, and the dispersal of knowledge. Therefore, comparing Twitter posts from different countries might explain certain differences found among them with regard to responding to the COVID-19 pandemic.

Assessing health-related behaviors (HRBs) on Twitter can help us understand the degree of awareness of the population using these preventive measures. It has been shown that there is an association between the characteristics of the content published in a certain geographic area and the rates of obesity and diabetes mellitus in that area [15]. In fact, a study showed that areas with the most tweets about physical activity or healthy foods (fruits, vegetables, etc) had lower obesity rates [16]. Twitter posts have also been analyzed to track behaviors related to the transmission of infectious diseases such as HIV [17]. Currently, multiple researchers are applying this methodology to better understand the reactions of the population, as well as raise awareness and promote compliance with health measures [18].

We conducted an observational retrospective study analyzing the content of mass media outlets posted on Twitter referring to COVID-19 during the early pandemic. Using Twitter, we sought to analyze the content posted by 30 major media outlets (17 from Spain and 13 from the United States) about COVID-19 during the first 6 months of the pandemic. Our two primary research aims were to (1) describe the extent to which major media outlets in the United States and Spain have tweeted about COVID-19 HRBs and determine if differences exist between major media outlets in the 2 countries and (2) determine the extent of user engagement in response to tweets about HRBs. We aimed to incorporate a cross-cultural perspective for several reasons. First, we sought to assess whether the media in Spain exhibited communication patterns similar to those in the United States. Furthermore, our hypothesis is that the beliefs and practices related to health may differ between the 2 countries because of political or cultural factors (such as differences in the health care system). In addition, the demographic characteristics of each country vary significantly, which can affect how the population experiences the pandemic. In Spain, a larger percentage of the population resides in multi-unit buildings where shared spaces are common, whereas individual houses are more prevalent in the United States.

## Methods

### Study Design and Overview

In this observational infodemiology study, we used concurrent collection and analysis of qualitative and quantitative data from tweets concerning public health measures relevant to the prevention and mitigation of the spread of the novel coronavirus posted by major media outlets in Spain and the United States.

### Data Collection

Tweets were drawn from a total of 30 major media outlets: 17 from Spain (Antena 3, La Sexta, TVE, Telecinco, cadena SER, cadena COPE, Onda Cero, ABC, El Pais, El Mundo, Europa Press, Noticias Cuatro, EFE, El Diario, La Vanguardia, Público, and Info Libre) and 13 from the United States (New York Times, Washington Post, Los Angeles Times, USA Today, The Chicago Tribune, New York Post, Wall Street Journal, Boston Globe, San Francisco Chronicle, MSNBC, CNN, ABC News, and CBS News). The 30 media outlets that we selected were general, had national coverage, had a large audience, and were among those with the highest social influence in their respective countries. We included different modalities, such as radio, newspapers, and television, to be more representative.

Our data collection strategy focused on identifying original tweets related to 4 HRBs: mask-wearing, physical distancing, quarantine or confinement, and hygiene. We included all original tweets posted from the Twitter accounts of the previously mentioned media outlets, thus avoiding the collection of tweets posted by other users, even if they were retweeted or mentioned by these media outlets, that referred to coronavirus (#corona, #coronavirus, #covid-19, #SARS-CoV2, and #2019-nCoV) and contained at least one of the following keywords (or their Spanish equivalents): #handwashing, #selfquarentine, #socialdistancing, #selfisolation, #masking, #mask, #disinfect, #disinfection, #socialgatherings, #sneez, #cough, #physical distancing, #facemask, #facecovering, #clothface, #lavadodemanos, #estornudo, #tos, #cuarentena, #distanciasocial, #distanciafísica, #aislamiento, #desinfectar, #desinfección, and #mascarilla. Original tweets posted between December 1, 2019, and May 31, 2020, were included, and data were collected on May 31, 2020. For each tweet, we extracted text, date, permanent link, and metadata. In addition, we collected user information for each of the media outlets, such as the number of followers and tweets posted with the mentioned hashtags. Finally, we extracted the number of likes and retweets generated by each tweet [19].

### Data Processing and Content Analysis

As described in our previous studies involving Twitter content analysis, our search tool Tweet Binder [20] allows access to 100% of all public tweets that match the search criteria (query). In contrast, other search engines based on Twitter’s free application programming interface (API) can only access a small sample [21,22]. Tweet Binder has its own data collection system that gathers all publicly available tweets on Twitter and retrieves both tweets and user information from Twitter’s API. Initially, the search tool scans the public section of Twitter to collect the IDs of tweets that match the search query. Subsequently, a call is made to the Twitter API to retrieve the tweet and user information. It does not directly access Twitter firehose itself.

**Figure 1:**
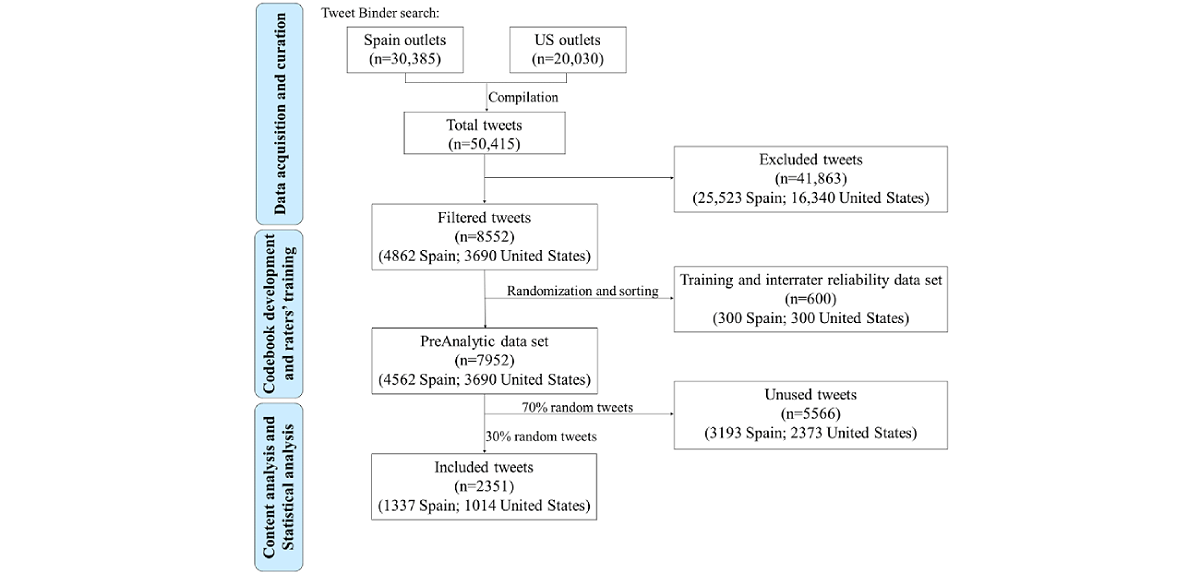
Flowchart.

Figure 1 summarizes the data collection and analysis steps, along with the number of tweets included and excluded in each step. First, we created a codebook based on our research question. There were 2 primary coders (MAA-M and FS). Coders first used a data set of 600 tweets to explore the content, generate codes, and obtain training. Discrepancies were discussed in regular team meetings. Training for coders was provided by the research team members (VPS and ART) experienced in content analysis and codebook development. After training, coders continued coding using the analytic data set. Interrater reliability was examined periodically to prevent rater drift. In a subset of 300 tweets, the interrater reliability averaged 91.7% (275/300) agreement for Spanish tweets and 83.3% (250/300) agreement for English tweets for different categories. The final codebook comprised six categories of HRB: (1) mask-wearing, (2) physical distancing, (3) handwashing, (4) quarantine or confinement, (5) disinfecting objects, and (6) multiple HRBs (any combination of the prior HRB categories). Examples of tweets are listed in Table 1.

**Table 1:** Examples of tweets of each health-related behavior (HRB). Tweets that mentioned >1 HRB were included in the Multiple HRBs category. Usernames and personal names were removed. All tweets reported here are in English (tweets in Spanish have been translated).

HRB	Examples of tweets
Quarantine or confinement	“Twitter is the first major U.S. corporation to strongly encourage its employees to work from home to avoid spreading coronavirus. ‘Beginning today, we are strongly encouraging all employees globally to work from home if they’re able.” “In R.I., 26 people have quarantined themselves while being monitored for coronavirus.” “Students and staff at a Winnetka school are being told to self-quarantine for 14 days, after a 7th-grader was diagnosed with a “probable” case of COVID-19.” “Olympic rugby player arrested after allegedly breaking Fiji’s coronavirus quarantine.”
Mask-wearing	“Globetrotting influencers combatting coronavirus with designer face masks.” “Fashion brands are making face masks, medical gowns for the coronavirus crisis.” “Woman wearing face mask attacked in possible coronavirus hate crime.” “Your hoarding masks could cost me my life—a doctor’s view from the coronavirus front lines.”
Social distancing	“DINING AT A DISTANCE: A Swedish couple has opened a ‘COVID-safe’ restaurant, with one table and one chair located in the middle of a meadow.” “Chicago-area Catholic churches are changing Mass practices to reduce risk of spreading coronavirus. Parishioners are being asked to avoid shaking hands during the Sign of Peace, using holy-water fonts or drinking communion wine.” “Despite state orders to social distance to prevent spread of coronavirus, many funerals in the ultra-Orthodox Jewish communities in Brooklyn continue to be large-scale events, prompting concern from leaders.”
Handwashing	“’Focus on slowing down the spread of COVID-19, the coronavirus. Did I mention: Wash. Your. Hands. Then wash them again,’ says epidemiologist Malia Jones.” “The best protection against coronavirus is washing your hands—but you have to do it properly: Wet your hands. Lather, making sure to get soap in all the nooks and crannies. Scrub for 20 seconds. Rinse and dry thoroughly.” “Coronavirus spawns viral TikTok dance about washing your hands.”
Disinfecting objects	“EPA releases list of disinfectant products approved for use against COVID-19 on surfaces-including multiple products from brands such as Clorox and Lysol.” “The Moscow Metro adopts stricter measures for disinfecting trains, as around 6000 metro carriages are being sterilized with UV light and disinfectant. The city is also starting construction on an infectious diseases hospital amid the coronavirus pandemic.” “Public spaces and elderly people’s homes sprayed with disinfectant by members of Spanish military as nation fights coronavirus pandemic.”
Multiple HRBs	“Cambridge is sending out ‘sound trucks’ to remind residents to stay 6 feet apart and wear masks during coronavirus pandemic.” “AHEAD: @[user] joins @[user] live to discuss how she turned her hit song ‘Get On Your Feet’ into a powerful message about wearing masks and staying home. Plus, why she’s bringing attention to minority communities disproportionately impacted by #coronavirus.” “People in many parts of the world are being asked to avoid crowds, limit travel and even work from home to help limit the spread of novel coronavirus, and satellite images suggest they’re heeding that advice.”

### Ethical Considerations

This study was initially reviewed by the Oregon Health & Science University Research Integrity Office and the institutional review board and was determined not to involve human participants. This study was approved to be conducted without continued institutional review board oversight and was compliant with the research ethics principles of the Declaration of Helsinki (7th revision, 2013).

### Statistical Analysis

First, we examined the prevalence of tweets with each HRB among tweets with at least one HRB by country, with *P*<.05 indicating a statistically significant difference in the proportion of a specific HRB in Spanish compared with US media sources. All further analyses were stratified by country, with Spain and the United States examined independently. Next, we calculated the measures of central tendency of the number of likes and retweets by HRB type. We assessed the distributions of the outcomes by tweet category and country, including calculating measures of central tendency and generating visualizations, such as histograms. The results showed substantial right skew for both the number of likes and retweets; thus, we presented the median (IQR) rather than mean (SD) and chose a Poisson distribution for our regression models.

Poisson mixed-effects regression models were run both unadjusted and with adjustment for media source number of followers, media source number of tweets, and follow-up time in days between tweet posting and the data collection date. We included media source as a random effect. Results were presented as the estimated counts of likes or retweets for tweets with each HRB compared with either (1) tweets with a different HRB or (2) tweets with no HRB. For ease of interpretation, estimated counts or average marginal effects were used instead of model coefficients. The reported *P* values indicate the significance of the association between the independent variable and the outcome (number of likes or retweets). Critical values for Bonferroni correction for multiple comparisons were calculated by dividing the α level (.05) by the number of hypotheses [6] and were applied to all results (critical value *P*<.008). All analyses were performed using Stata (version 16; StataCorp).

## Results

### Distribution of Tweets by HRBs

We collected all the tweets that included a hashtag mentioning COVID-19 (in different ways: #coronavirus, #covid-19, #SARS-CoV2, and #2019-nCoV) and any of the HRBs mentioned previously (including Spanish equivalents). That is, the tweet had to mention COVID and at least one HRB to be collected. With these search criteria, we collected 50,415 tweets but excluded 41,863 because they had nothing to do with any HRB. At that time, it was very common to include these types of hashtags in tweets, even though the content of the tweets had nothing to do with health issues, so we decided to exclude them. Of the remaining 8552 tweets, we randomly selected 600 to design the codebook and train the raters. Finally, 7952 were left, and 30% (2386) were randomly selected for manual analysis.

The number of HRB posts varied considerably among the analyzed mass media accounts (Multimedia Appendix 1). Overall, mass media outlets from Spain showed the largest number of posts. In particular, EFE Noticias, Antena 3, and TVE y La Sexta were the most active media outlets in Spain, whereas CNN, ABC, and New York Post were the most active outlets in the United States. Among the outlets that posted >100 tweets, the New York Post and ABC had the highest proportion of HRB tweets, 78.8% (93/118) and 70.1% (109/154) respectively, whereas TVE and Antena 3 had the lowest proportion, 58.2% (89/153) and 59.4% (95/160) respectively.

As shown in the flowchart in Figure 1, of the 50,415 tweets collected, 2351 were included in the content analysis, and 1479 of them (62.91%) mentioned at least one HRB. As shown in Table 2, the proportion of tweets mentioning HRBs was significantly different between the 2 countries (*P*=.006); 60.51% (809/1337) of all tweets related to COVID-19 posted by media outlets from Spain and 66.07% (670/1014) of tweets posted by US outlets contained at least one HRB (Table 2). In both countries, the distribution of tweets across different categories of HRB was heterogeneous. Quarantine or confinement-related tweets accounted for the highest proportion of tweets in both countries, 48.7% (394/809) in Spain and 49.7% (333/670) in the United States), followed by tweets related to masks and social distancing. In contrast, the least frequent HRB categories were disinfecting objects in Spain, 6.9% (56/809), and handwashing in the United States, 9.1% (61/670). There was no significant difference in the proportion of tweets mentioning each HRB between major Spanish and US media outlets.

**Table 2:** Descriptive characteristics of the tweets regarding health-related behaviors (HRBs) for the prevention of COVID-19, by category^a^ and country.

		Country			*P* value
		Spain	United States		
All COVID-19 tweets, n		1337	1014	N/A^b^	
Tweets with 0 HRB, n (%)		528 (39.5)	344 (33.9)	.006	
Tweets with ≥1 HRB, n (%)		809 (60.5)	670 (66.1)	N/A	
**Among COVID-19 tweets with ≥1 HRB, n (%)**					
	Quarantine or confinement	394 (48.7)	333 (49.7)	.70	
	Masks	285 (35.2)	227 (33.9)	.59	
	Social distancing	129 (16)	84 (12.5)	.06	
	Handwashing	80 (9.9)	61 (9.1)	.61	
	Disinfecting objects	56 (6.9)	65 (9.7)	.05	
	Multiple HRBs	98 (12.1)	70 (10.5)	.32	

^a^Categories are not mutually exclusive; tweets with multiple HRBs are counted in those categories as well as in the Multiple HRBs category. Subgroup restricted to tweets with ≥1 HRB and >3 days between tweet posts and data collection date.

^b^N/A: not applicable.

### Engagement Metrics by HRB and by Country

We investigated engagement with tweets posted by Spanish and US media outlets from social media users by analyzing the number of likes and retweets received. Tweets mentioning at least one HRB received a similar number of likes and retweets as those tweets that did not mention an HRB, as shown in Table 3. In both Spain and the United States, the median number of likes received by each tweet was higher than the median number of retweets. Among tweets with at least one HRB, disinfecting objects had a median (IQR) of 197 (63-486) likes and 90 (33-246) retweets, which constitutes the highest for tweets from US media outlets, and it is twice the number achieved by quarantine or confinement-related tweets (Table 3). Among those posted by Spanish media, mask- and handwashing-related tweets had the highest median number of likes (22 and 21, respectively), whereas social distancing-related tweets had the lowest median number of likes. In contrast, all HRBs had a similar median number of retweets (11 or 12).

Results from mixed Poisson regression analyses in tweets with clustering by media source are presented in Table 4 (Spain) and Table 5 (United States). In adjusted models, tweets posted by mass media outlets from Spain that mentioned social distancing or mentioned disinfecting objects had a significantly lower predicted count of likes compared with tweets mentioning a different HRB (*P*=.02 and *P*=.01, respectively) or tweets related to COVID-19 but not mentioning any HRB at all (*P*=.01 and *P*=.005, respectively). Tweets mentioning multiple HRBs also had a significantly lower count of predicted likes than tweets mentioning just 1 HRB (*P*<.001) or did not mention any HRB (*P*<.001). In respect of retweets, disinfecting objects and tweets mentioning multiple HRBs had a significantly lower predicted number of retweets than tweets mentioning other HRB’s or not mentioning any HRB. Other associations that were not significant (*P*<.05 are presented in Table 4.

In regard to tweets posted by US media outlets, in adjusted models those mentioning quarantine or confinement or disinfecting objects had a significantly lower predicted number of likes compared with tweets mentioning a different HRB (*P*<.001), whereas mask- and handwashing-related tweets had a significantly greater predicted number of likes (*P*=.04 and *P*=.02, respectively; Table 5). When compared with tweets related to COVID-19 but not mentioning an HRB, those tweets related to quarantine or confinement or to social distancing had a significantly lower predicted number of likes (*P*=.01 and *P*=.005, respectively). With respect to retweets, quarantine or confinement had a significantly lower predicted number of retweets than tweets mentioning other HRB’s (*P*<.001), whereas those mentioning handwashing had a greater probability of being retweeted (*P*=.006). When compared with tweets related to COVID-19 not mentioning an HRB, those tweets related to quarantine or confinement, social distancing, or multiple HRBs had a significantly lower predicted number of retweets (*P*<.001).

**Table 3:** Distribution of likes and retweets per category, by country.

Category		Spain								United States				
		Value, n			Likes, median (IQR)		Retweets, median (IQR)		Value, n			Likes, median (IQR)		Retweets, median (IQR)
All COVID-19 tweets		1337			19 (8-44)		11 (5-28)		1014			109 (36-316)		54 (18-137)
Tweets with 0 HRB^a^		528			18 (8-41)		12 (5-26)		344			105 (35-294)		57 (19-139)
Tweets with ≥1 HRB		809			20 (8-47)		11 (5-30)		670			110 (37-321)		51 (18-135)
**Among COVID-19 tweets with ≥1 HRB**														
	Quarantine or confinement		394	17 (7-39)		11 (5-28)		333			88 (31-262)		45 (16-102)	
	Masks		285	22 (10-53)		12 (5-35)		227			127 (41-588)		57 (20-183)	
	Social distancing		129	16 (9-37)		11 (5-23)		84			171 (44-390)		62 (17-127)	
	Handwashing		80	21 (8-56)		11 (4-44)		61			118 (58-447)		59 (24-179)	
	Disinfecting objects		56	18 (10-59)		12 (6-48)		65			197 (63-486)		90 (33-246)	
	Multiple HRBs		98	13 (7-32)		11 (4-25)		70			191 (58-358)		66 (28-175)	

^a^HRB: health-related behavior.

**Table 4:** Average predicted number of likes by presence or absence of category in Spain. Results presented as predicted mean (95% CI) from adjusted Poisson models with clustering for media source. Adjustment variables include source number of followers, source number of tweets, and days from tweet posting to data collection date.

			Tweet with specific HRB^a^ versus tweet with different HRB (n=809)							Tweet with specific HRB versus tweet with no HRB						
			Tweet with a different HRB, mean (95% CI)		Presence of HRB, mean (95% CI)		*P* value^b^		Model, n			Tweet with no HRB, mean (95% CI)		Presence of HRB, mean (95% CI)		*P* value^b^
**Likes**																
	Quarantine or confinement	96.8 (42.5-151.1)		67.3 (51.4-83.1)		.19		922			80.5 (60.1-100.9)		67.3 (51.8-79.6)		.09	
	Masks	65.4 (51.4-79.3)		115.7 (26.9-204.5)		.16		813			79.7 (59.9-99.5)		119.1 (31.4-206.7)		.18	
	Social distancing	90.2 (51.2-129.2)		43.5 (29.1-57.8)		.02		657			80.1 (58.5-101.6)		45.3 (32.0-58.6)		.01	
	Handwashing	85.5 (48.8-122.11)		62.0 (37.1-86.9)		.36		608			82.3 (59.9-104.6)		63.2 (43.2-83.2)		.24	
	Disinfecting objects	86.5 (51.5-121.4)		44.7 (30.6-58.9)		.01		584			82.3 (60.4-104.3)		49.2 (32.4-65.9)		.005	
	Multiple HRBs	89.1 (52.8-125.4)		37.7 (28.3-47.1)		<.001		626			80.8 (59.5-102.0)		38.8 (28.2-49.4)		<.001	
**Retweets**																
	Quarantine or confinement	60.1 (20.2-100.0)		48.0 (35.8-60.2)		.49		922			53.6 (38.7-68.6)		45.9 (35.8-55.9)		.22	
	Masks	46.5 (35.7-57.2)		69.4 (3.4-135.4)		.43		813			52.2 (37.9-66.4)		71.5 (5.8-137.3)		.42	
	Social distancing	57.6 (28.3-86.9)		36.5 (23.4-49.7)		.25		657			52.4 (36.6-68.2)		38.0 (24.9-51.1)		.25	
	Handwashing	56.1 (29.2-82.9)		41.1 (27.6-54.6)		.36		608			54.3 (38.6-69.9)		42.2 (30.7-53.7)		.20	
	Disinfecting objects	56.7 (30.8-82.7)		30.3 (18.7-42.0)		.04		584			54.1 (38.7-69.6)		34.5 (20.9-48.2)		.03	
	Multiple HRBs	57.7 (30.6-84.9)		29.9 (21.2-38.6)		.04		626			53.0 (37.9-68.2)		30.8 (21.3-40.4)		.01	

^a^HRB: health-related behavior.

^b^*P* value is for the association (independent variable coefficient) between the presence or absence of a category and the number of likes it received.

**Table 5:** Average predicted number of likes by presence or absence of category in United States. Results presented as predicted mean (95% CI) from adjusted Poisson models with clustering for media source. Adjustment variables include source number of followers, source number of tweets, and days from tweet posting to data collection date.

		Tweet with specific HRB^a^ versus tweet with different HRB (n=669)				Tweet with specific HRB versus tweet with no HRB			
		Tweet with a different HRB, mean (95% CI)	Presence of HRB, mean (95% CI)	*P* value^b^	Model, n		Tweet with no HRB, mean (95% CI)	Presence of HRB, mean (95% CI)	*P* value^b^
**Likes**									
	Quarantine or confinement	709.2 (404.7-1013.7)	331.7 (250.5-414.4)	<.001	677		510.3 (444.9-575.7)	351.3 (259.9-442.7)	.01
	Masks	467.1 (288.3-645.9)	620.0 (369.8-870.2)	.04	571		523.4 (439.0-607.8)	590.0 (333.0-847.0)	.66
	Social distancing	536.2 (303.7-768.7)	395.3 (258.1-532.4)	.41	428		540.9 (480.5-601.3)	398.2 (314.9-481.5)	.005
	Handwashing	448.3 (347.4-551.0)	1154.6 (46.9-2262.4)	.02	405		515.3 (427.5-603.1)	1357.8 (90.2-2625.4)	.06
	Disinfecting objects	542.7 (335.5-750.0)	305.8 (250.1-361.5)	<.001	409		539.9 (478.5-601.3)	306.3 (137.3-475.4)	.06
	Multiple HRBs	535.4 (319.4-751.3)	364.6 (273.5-455.7)	.20	414		524.0 (458.9-589.2)	393.0 (282.8-503.1)	.08
**Retweets**									
	Quarantine or confinement	263.4 (143.0-383.9)	120.7 (83.5-157.9)	<.001	677		223.1 (197.2-249.1)	129.3 (89.7-169.0)	<.001
	Masks	177.8 (102.8-252.8)	212.8 (119.9-305.7)	.26	571		226.6 (192.7-260.4)	201.1 (104.2-298.1)	.68
	Social distancing	198.4 (108.4-288.4)	127.8 (94.8-160.8)	.18	428		234.2 (208.2-260.3)	122 (102.7-142.8)	<.001
	Handwashing	159.6 (115.8-203.5)	463.0 (30.7-895.2)	.006	405		224.9 (190.6-259.2)	525.4 (60.3-990.5)	.08
	Disinfecting objects	192.3 (110.0-274.7)	160.1 (134.5-185.7)	.32	409		233.3 (206.9-259.8)	153.9 (79.9-227.8)	.10
	Multiple HRBs	195.8 (111.3-280.2)	134.3 (110.5-158.2)	.12	414		228.3 (200.2-256.4)	141.1 (111.0-171.1)	<.001

^a^HRB: health-related behavior.

^b^*P* value is for the association (independent variable coefficient) between the presence or absence of a category and the number of likes it received.

## Discussion

### Principal Findings

In this study, we found that major media outlets from Spain and the United States, when posting information on Twitter related to COVID-19, mentioned an HRB in the majority of their tweets, both focusing on quarantine or confinement and masks. Twitter users from both countries showed similar engagement in tweets mentioning an HRB compared with those that did not mention an HRB. Remarkably, the engagement of users following media outlets from Spain was more equally distributed among different HRBs. Furthermore, in Spanish tweets, none of the HRB tweets had a higher probability of being liked or retweeted than others. However, tweets mentioning handwashing or masks had greater probabilities of being liked than tweets mentioning a different HRB or not mentioning any of them when posted from US media outlets. Finally, we observed that media outlets from Spain differed in their Twitter posting patterns quantitatively and qualitatively from US media outlets.

It has previously been shown that entertainment media and social media play a critical role in the behaviors of individuals and have the potential to influence awareness, which is important because adhering to health recommendations is considered a very relevant element for the prevention of COVID-19 infection and overcoming the pandemic [23-25]. Certain health recommendations have been changed since the outbreak of the pandemic [26]. However, majority of health professionals and institutions have promoted some HRBs since the early stages of the pandemic. Social media platforms such as Twitter are increasingly being leveraged by researchers for surveillance and to explore complex social issues, such as perceptions of the public on HRBs, including masks, handwashing, social distancing, and vaccines [27-29]. Furthermore, recognized socially influential agents, such as media outlets or politicians, use Twitter as a dissemination tool for their information, including COVID-19–related news [30]. Social media has become the main source of COVID-19–related information for many people [31]. When media outlets share information, their influence is enormous, particularly in situations such as a pandemic [32,33]. Thus, it is important to analyze the impact of this information on society because exposure to misinformation has been associated with psychological distress, poorer COVID-19 knowledge, and lower adoption of preventive behaviors [34].

### Communication Media and HRBs

Our data show that the number of tweets posted by major media outlets from Spain and the United States regarding HRBs is high overall. Interestingly, the number of posts was not homogeneously distributed among the different categories, with quarantine or confinement and masks receiving the highest number of tweets. These results point in the same direction as previous reports, which also found that a great variety of COVID-19–related human behaviors have been discussed on social media, with masks and sheltering in place prevailing over others [35,36]. In fact, Americans initially posted about China, but once COVID-19 became a reality in the United States, their social media posts started to focus on US-centered issues, such as lockdown or stay-at-home recommendations [37]. Nonetheless, those following Twitter accounts of major US media outlets were more interested in handwashing, whereas those following Spanish outlets did not show such a preference.

Several reasons may explain the differences found in the priorities or interests of the media and Twitter users, as well as the differences found between users following media outlets from Spain and the United States. First, it may be in the best interest of media outlets and politicians to focus on issues related to masks (shortage, logistics organization of distribution, legislation, etc) rather than on promoting less controversial matters such as handwashing. Second, the great mediatic and social impact generated by quarantines and confinements in comparison with other measures established to prevent the spread of COVID-19 may explain why this HRB, in particular, is so present in tweets posted by media outlets [38,39]. Third, wide sectors of society, such as political parties in the opposition, may push the media to speak and try to generate buzz around quarantine or confinement and masks rather than promoting healthy habits that are not controversial, such as handwashing. Fourth, many authorities may be especially active in promoting confinement through press news because of its implications. In fact, politicians’ announcements related to confinements and quarantines have been disseminated through media outlets on social media accounts. In addition, several studies have suggested that political parties and big companies have a strong influence on the agenda-setting of media outlets and on the information that they distribute. All of these facts may have contributed to our observed prominence of quarantine or confinement and mask tweets, as compared with handwashing, despite the fact that the latter generated more engagement among Twitter users.

In our study, differences were found in the probability of tweets being liked or retweeted according to each HRB. However, it is relevant to note that all HRB tweets achieved a median number of likes and retweets higher than those found in previous articles for tweets posted by US media outlets on diseases with high prevalence and morbidity (such as cancer, Parkinson, depression, and osteoporosis) or on tweets related to other preventive medical measures, such as contraceptives [21,40]. Moreover, it is important to note that these differences in the probabilities of a tweet being liked or retweeted were more pronounced in tweets posted by US media outlets. One possible explanation could be that US media outlets not only have a greater number of followers but also a more international audience. This greater diversity among followers may contribute to greater polarization in their interests. Nevertheless, cultural differences among countries regarding public perceptions and preventive behaviors during the COVID-19 pandemic have been previously described [41]. Furthermore, the relative weight assigned to each HRB by the media outlets, as defined by the percentage of tweets received, was not related to the retweets and likes generated by Twitter users. That is, the HRB that gathered the most attention was handwashing, whereas tweets mentioning multiple HRBs did not generate much attention despite prestigious studies showing strong evidence of each of the HRB mentioned. This finding could be explained by the fact that tweets are short in nature and may be easier to capture user’s attention if only focusing on a specific HRB. In addition, Twitter users might have been prone to promoting handwashing, because the benefits of doing so are strongly supported by scientific data and have never been questioned. Furthermore, handwashing, in contrast to masks or quarantine or confinement, has no political connotations, thus allowing Twitter users to share handwashing posts without publicly declaring their political preferences [42]. Moreover, tweets mentioning disinfecting objects despite President Trump’s declarations in this regard and all the controversies generated had lower probabilities of being retweeted or liked than tweets mentioning a different HRB.

In addition, we analyzed the proportion of tweets posted by each of the media outlets analyzed, mentioning each HRB. Our data showed that most media outlets, with only 1 exception, focused on quarantine or confinement and masks. Thus, bias in the information related to HRB was not detected. This may indicate that media outlets share common interests. Nonetheless, it is important to highlight that certain differences were observed between them.

The important role that media outlets play in generating popular opinion is well known. Thus, our results suggest that health promotion is not as relevant as generating controversy for media outlets. This is worrying given that measures such as washing hands or maintaining physical distancing are as important as wearing masks or complying with quarantine or confinement, despite the latter being the object of more controversy. The adoption of all HRB is desirable to prevent COVID-19 infection. However, according to our results, controversial measures attract more attention from the media but not from Twitter users.

### Limitations

It should be noted that this study has limitations. First, the relevance of Twitter as a social interest marker remains controversial. In addition, the lack of data regarding the geographic location of Twitter users is a limitation in interpreting engagement. Second, the analyzed media outlets do not necessarily reflect the posting pattern of all the press and might have a different set of priorities. Third, our Twitter data were collected according to our selected keywords; thus, we might have missed tweets using different keywords despite discussing the same topic. Fourth, content analysis implies a certain degree of subjectivity. To address this issue, the study comprised a series of steps: initial review, the design of a codebook through a comprehensive process, and the testing of a coder agreement.

### Conclusions

To our knowledge, this study is the first to compare media outlet posts related to COVID-19 HRBs from 2 different countries. Media outlets provided more content related to quarantine or confinement and masks, whereas Twitter users, especially those following US media outlets, showed greater engagement with handwashing. Moreover, tweets mentioning multiple HRBs did not result in as much engagement from the Twitter community as those mentioning only 1 HRB. We believe that this finding may have been influenced by the nature of Twitter. Understanding health communication on social media is necessary to design appropriate public health campaigns that might contribute to reducing the rates of contagion and ultimately overcome the COVID-19 pandemic. Future studies could expand the current research by assessing the impact of media publications on the evolution of the pandemic.

## Appendix

Multimedia Appendix 1 Number of tweets by media outlet, by country. Results presented as n (%) of health-related behavior tweets.

## Abbreviations

API: application programming interface
HRB: health-related behavior
