# Supplementary material for: Content and User Engagement of Health-Related Behavior Tweets Posted by Mass Media Outlets From Spain and the United States Early in the COVID-19 Pandemic: Observational Infodemiology Study
Source: JMIR Infodemiology. 2023 Aug 22;3:e43685. doi: 10.2196/43685 (PMC10445660; doi:10.2196/43685)
Supplement: Multimedia Appendix 1 [file infodemiology_v3i1e43685_app1.docx]

**Supplemental Table (Appendix 1).** Number of tweets by media outlet, by country. Results presented as n (% of HRB tweets)

**SPAIN**

|  | All COVID tweets |  | ALL HRB tweets | Quarantine | Masks | Social distancing | Hand washing | Disinfecting objects | Multiple HRBs |
| --- | --- | --- | --- | --- | --- | --- | --- | --- | --- |
| EFE Noticias | 204 |  | 140 | 85 (60.7) | 39 (27.9) | 21 (15.0) | 6 (4.3) | 4 (2.9) | 11 (7.9) |
| Antena 3 | 160 |  | 95 | 39 (41.1) | 41 (43.2) | 18 (18.9) | 3 (3.2) | 4 (4.2) | 8 (8.4) |
| TVE | 153 |  | 89 | 38 (42.7) | 39 (43.8) | 18 (20.2) | 16 (18.0) | 7 (7.9) | 18 (20.2) |
| LaSexta | 114 |  | 69 | 34 (49.3) | 29 (42.0) | 6 (8.7) | 8 (11.6) | 4 (5.8) | 11 (15.9) |
| El Pais | 92 |  | 52 | 22 (42.3) | 18 (34.6) | 7 (13.5) | 7 13.5) | 6 (11.5) | 6 (11.5) |
| ABC ES | 82 |  | 53 | 25 (47.2) | 24 (45.3) | 8 (15.1) | 3 (5.7) | 4 (7.5) | 4 (7.5) |
| Telecinco | 81 |  | 40 | 16 (40.0) | 11 (27.5) | 7 (17.5) | 7 (17.5) | 6 (15.0) | 5 (12.5) |
| El Diario Es | 78 |  | 41 | 20 (48.8) | 13 (31.7) | 8 (19.5) | 4 (9.8) | 1 (2.4) | 4 (9.8) |
| Europa Press | 70 |  | 52 | 24 (46.2) | 14 (26.9) | 8 (15.4) | 7 (13.5) | 8 (15.4) | 8 (15.4) |
| Cadena COPE | 66 |  | 44 | 30 (68.2) | 8 (18.2) | 5 (11.4) | 4 (9.1) | 1 (2.3) | 4 (9.1) |
| La Vanguardia | 65 |  | 38 | 15 (39.5) | 16 (42.1) | 10 (26.3) | 4 (10.5) | 3 (7.9) | 8 (21.2) |
| El Mundo | 59 |  | 37 | 20 (54.1) | 12 (32.4) | 6 (16.2) | 3 (8.1) | 2 (5.4) | 4 (10.8) |
| Cadena SER | 44 |  | 20 | 9 (45.0) | 9 (45.0) | 4 (20.0) | 5 (25.0) | 3 (15.0) | 6 (30.0) |
| Publico Es | 26 |  | 15 | 8 (53.3) | 3 (20.0) | 2 (13.3) | 1 (6.7) | 1 (6.7) | 0 |
| Onda Cero | 17 |  | 12 | 4 (33.3) | 6 (50.0) | 1 (8.3) | 1 (8.3) | 0 | 0 |
| Info Libre | 15 |  | 7 | 4 (57.1) | 2 (28.6) | 0 | 0 | 0 | 1 (14.3) |
| Noticias Cuatro | 11 |  | 5 | 1 (20.0) | 1 (20.0) | 0 | 1 (20.0) | 2 (40.0) | 0 |
| Total | 1,337 |  | 809 | 394 (48.7) | 285 (35.2) | 129 (16.0) | 80 (9.9) | 56 (6.9) | 98 (12.1) |

**USA**

|  | All COVID tweets |  | ALL HRB tweets | Quarantine | Masks | Social distancing | Hand washing | Disinfecting objects | Multiple HRBs |
| --- | --- | --- | --- | --- | --- | --- | --- | --- | --- |
| CNN | 172 |  | 114 | 63 (55.3) | 34 (29.8) | 17 (14.9) | 14 (12.3) | 12 (10.5) | 14 (12.3) |
| ABC | 154 |  | 109 | 49 (45.0) | 26 (23.9) | 19 (17.4) | 11 (10.1) | 20 (18.5) | 13 (12.0) |
| New York Post | 118 |  | 93 | 44 (47.3) | 37 (39.8) | 3 (3.2) | 10 (10.8) | 4 (4.3) | 4 (4.3) |
| LA Times | 93 |  | 69 | 36 (52.2) | 27 (39.1) | 8 (11.6) | 5 (7.2) | 3 (4.3) | 9 (13.0) |
| MSNBC | 73 |  | 36 | 13 (36.1) | 19 (52.8) | 5 (13.9) | 3 (8.3) | 3 (8.3) | 3 (8.3) |
| New York Times | 57 |  | 40 | 24 (60.0) | 15 (37.5) | 4 (10.0) | 1 (2.5) | 3 (7.5) | 6 (15.0) |
| CBS News | 57 |  | 35 | 15 (42.9) | 16 (45.7) | 6 (17.1) | 3 (8.6) | 4 (11.4) | 6 (17.1) |
| Washington Post | 56 |  | 28 | 12 (42.9) | 13 (46.4) | 1 (3.6) | 2 (7.1) | 1 (3.6) | 1 (3.6) |
| SF Chronicle | 56 |  | 28 | 20 (71.4) | 7 (25.0) | 2 (7.1) | 3 (10.7) | 2 (7.1) | 4 (14.3) |
| Chicago Tribune | 53 |  | 31 | 13 (41.9) | 12 (38.7) | 5 (16.1) | 2 (6.5) | 4 (12.9) | 3 (9.7) |
| USA Today | 46 |  | 34 | 16 (47.1) | 7 (20.6) | 4 (11.8) | 4 (11.8) | 6 (17.6) | 2 (5.9) |
| Wall Street Journal | 44 |  | 28 | 18 (64.3) | 5 (17.9) | 3 (10.7) | 1 (3.6) | 2 (7.1) | 1 (3.6) |
| Boston Globe | 35 |  | 25 | 10 (40.0) | 9 (36.0) | 7 (28.0) | 2 (8.0) | 1 (4.0) | 4 (16.0) |
| Total | 1,014 |  | 670 | 333 (49.7) | 227 (33.9) | 84 (12.5) | 61 (9.1) | 65 (9.7) | 70 (10.5) |
